# Is Hematopoietic Clonality of Indetermined Potential a Risk Factor for Pulmonary Embolism?

**DOI:** 10.1055/s-0041-1733856

**Published:** 2021-08-17

**Authors:** S. Soudet, G. Jedraszak, O. Evrard, J.P. Marolleau, L. Garcon, M.A. Sevestre Pietri

**Affiliations:** 1 Department of Vascular Medicine, CHU Amiens Picardie, Amiens, France; 2 EA 7516 CHIMERE, Université Picardie Jules Verne, Amiens, France; 3 Department of Genetic, CHU Amiens Picardie, Amiens, France; 4 EA 4666 HEMATIM, Université Picardie Jules Verne, Amiens, France

**Keywords:** gene mutations, pulmonary embolism, venous thrombosis

## Abstract

**Background** Unprovoked pulmonary embolism (uPE) is a severe and frequent condition. Identification of new risk factors is mandatory to identify patients that would benefit from a long-term treatment. Clonal hematopoiesis of indeterminate potential (CHIP) is defined by the acquisition of somatic mutations that drive clonal expansion in the absence of cytopenia. Its prevalence is estimated of 5% in the population above 65 years. Since inflammation and endothelial dysfunction may share a pathophysiological pathway(1), we hypothesized that CHIP, may be a risk factor for uPE.

**Methods** We conducted a pilot retrospective observational study. Patients with iPE between 18 to 65 years old were included. PE was considered as unprovoked, when no transient nor persistant risk factor was present and when thrombophilia testing was negative. We excluded documented atherosclerosis, personal or familial history of VTE and presence of cytopenias. CHIP proportion in uPE patients were analyzed using next generation sequencing of the coding sequence of a custom panel composed by *DNMT3A, ASXL1, SF3B1, TET2* and *TP 53* .

**Results** Upon 61 patients with uPE consecutively included, a total of 19 somatic mutations were found in 12 patients (20%) IC95% [10 - 20]. 15 mutations were found in *DNMT3A* gene, 3 in *ASXL1* and one in *TET2* . There was no diference in terms of age, PE location, DVT presence and risk stratification in CHIP carriers and non carriers.

**Conclusion** We report for the first time, the presence of high rates of CHIP in patients presenting with uPE. Thus, CHIP may be a new risk factor for VTE. These results need to be confirmed in an ongoing prospective case-control study including more patients and using a more diverse gene panel to better determine CHIP incidence in uPE.

## Introduction

Idiopathic pulmonary embolism (iPE), defined by a proximal pulmonary embolism (PE) in the absence of major or minor risk factors, is frequent, accounting for half of reported PEs. 1 It is also a severe condition, with a 10% mortality risk and a higher rate of recurrence, reaching 10% per year according to a large prospective study. 1 It requires long term anticoagulant treatment as shown in the PADIS-PE study. 2 Recently, guidelines recommend the maintenance of a long-term anticoagulant treatment in idiopathic VTE. 3 4 5 However, iPE is an heterogeneous state and characterization of new risk factors is mandatory to identify patients needing a long term treatment to avoid reccurence. Numerous scores have already been proposed to predict the individual risk of recurrence 2 3 4 but there is still a need to decipher better pathophysiological mechanisms involved in iPE, as highlighted recently by the American Heart Association and the International Society of Thrombosis and Haemostasis. 5

Clonal hematopoiesis of indeterminate potential (CHIP) is defined as the acquisition of somatic mutations that drive clonal expansion in the absence of cytopenia and dysplastic hematopoiesis. 6 These mutations occur in many different genes involved in hematopoietic stem cells physiology, including signaling pathway, epigenetic regulation, transcription control, DNA repair or splicing control; the most frequent ones being *DNMT3A* , *TET2* and *ASXL1* . CHIP is frequent in the elderly, occurring in 9.5% of cases after 70 years old. 7 Before 65 years old, its prevalence is estimated of 5%. However, its incidence varies, depending on the sensitivity of the sequencing technologies to detect low variant allele fraction (VAF), most studies using currently a threshold of 2% 8 or 1%. 9 In addition to its well-identified risk of hematological malignancies, CHIP is now considered as a risk factor for cardiovascular diseases (CVD) as described in recent reviews. Jaiswal et al reported a link between CHIP and premature atherosclerosis 8 possibly related to pro-inflammatory interactions between clonally derived leukocytes and vascular endothelial models. 10 11 Indeed, attention has mainly been focused so far on proposed mechanisms of accelerated inflammation-driven atherosclerosis and increased thrombosis risk through altered function of innate immune cells. Moreover, a deep vein stenosis murine model showed a reduction of thrombosis by inhibition in mice with conditional knocked-in of *JAK2 ^V617F^* mutation. 12

The association between venous and arterial thrombosis is still a matter of debate. 13 Some risks factors are shared by artherosclerosis and venous thromboembolism (VTE) such as age, male sex and obesity. Moreover, patients with VTE are at higher risk of subsequent cardiovascular events, particularly in case of iPE. 14 15 Since inflammation and endothelial dysfunction could share pathophysiological pathways, 16 we hypothesized that CHIP, in addition to its association with CVD, is also a risk factor for iPE.

## Methods

We conducted a pilot retrospective monocentric observational study in patients with iPE followed in the university hospital of Amiens-Picardie between January 2018 and December 2019. All patients gave their informed consent according to Helsinki declaration and this non interventional study was approved by local ethic committee. Since the expected incidence of CHIP is low in younger patients, we restricted our analysis to patients with iPE between 18 to 65 years of age, after a first episode of PE documented by computed tomography pulmonary angiography. We hypothesize that identification of high proportion of CHIP in this population would suggest an association between CHIP and uPE. PE was considered as unprovoked when no transient or persistant risk factor were present and when extensive thrombophilia testing was negative, including Factor V Leiden and prothrombin 20210A mutations, antithrombin, C and S protein activity, anti cardiolipin, anti Beta2GP1 antibodies and lupus anticoagulant. Search for driver mutation of myeloproliferative neoplasms ( *i.e* JAK ^V617F^ , CalR and MPL mutations) was systematically performed. All patients underwent complete Doppler ultrasound of lower limb at diagnosis. Patients with documented atherosclerosis, previous VTE, any positive thrombophilia, minor or major risk factor, familial history of VTE, presence of cytopenias, presence of any driven mutation of myeloproliferative neoplasms were excluded. Risk stratification of patients with PE was based on clinical symptoms, hemodynamic instability and right ventricle dysfunction. Patients were classified as low, intermediate or high risk of early death as recommended in recent guidelines. 17

For CHIP analysis, DNA samples were obtained from peripheral blood cells and were analyzed using next generation sequencing of the coding sequence of a custom panel composed by *DNMT3A, ASXL1, SF3B1, TET2* and *TP 53* . We chose a limited panel due to the high frequency of these genes in CHIP 18

Sample preparation and capture of regions of interest were performed using SureSelect XHTS low input protocol with molecular barcoding technology (Agilent Technologies®). Sequencing was performed on Illumina Miseq platform with an objective of 1000X of depth. Bioinformatic analyses were performed by Alissa Align&Call software (Alissa – Agilent Technologies®) using set-up optimized to detect variant with more than 1% allele frequency. Variant interpretation was based on the Catalog of Somatic Mutations in Cancer (COSMIC; http://cancer.sanger.ac.uk/cancergenome/projects/cosmic ) and literature data. CHIP mutation was defined as the presence of a mutation in the coding region of a gene of the panel, in the absence of blood count abnormality or hematologic malignancy and with a variation VAF above 1%.

Statistical analysis was performed with R software, using Student's *t* -test with a statistical difference at α = 0.05.

## Results

A total of 61 patients, 8 women (13.1%) and 53 men (87%), meeting the inclusion criteria were consecutively included, with a median age of 54 years [47–59.5]. 60% of patients had proximal deep vein thrombosis at diagnosis. 30 patients (49.2%) had a low risk PE, 20 patients (32.3%) had an intermediate low risk PE and 11 patients (19.5%) had an intermediate high risk PE. Median platelets numeration was 247. 10 ^9^ per liter [170–250], median hematocrit was 43% [39–45,2] and median white blood count (WBC) was 5.5.10 9 /L [5 - 7]. There was also no difference in other hemogram parameters as red blood distribution or mean corpuscular volume. The median follow-up was 2 years [1.5 - 2]. All patients were treated with direct oral anticoagulants, with recommended regimen according to international guidelines, 40% ( *n* = 24) received rivaroxaban and 60% ( *n* = 37) apixaban. During follow up, no VTE reccurence was noticed and no pulmonary hypertension was diagnosed. All patients were alive at the last follow-up endpoint. For all of them, hematological parameters remained in the normal range and no hematological malignancy nor solid cancer were diagnosed during the whole follow-up.

Considering a VAF of 1%, 19 somatic mutations were found in 12 patients (20%) 95%IC[10–30]. With a VAF of 2%, somatic mutations was present in 6 patient (10%). Patients characteristics and acquired mutations are detailed in Table 1 . In patients with CHIP, median age was 59.5 years [56.25–65]. Two of them were women (16.7%) and 10 were men (83.3%). There was no difference in terms of age, PE location, DVT presence and risk stratification in CHIP carriers and non carriers. Hematological parameters were also not discriminative between these two populations and are shown in Table 2 .

**Table 1:** Characteristics of CHIP carriers

Age	Sex	PE location	DVT	Mutated gene	VAF(%)
58	M	Segmental bilateral	Proximal	ASXL1	1,2
37	M	Segmental bilateral	No	DNMT3A	1,3
65	M	Proximal Bilateral	No	DNMT3A	26
				TET2	26
66	M	Proximal bilateral	Proximal	DNMT3A	7,8
47	M	Proximal bilateral	No	DNMT3A	5
58	M	Proximal bilateral	Proximal	ASXL1	1,4
57	F	Proximal bilateral	No	DNMT3A	1,5
				DNMT3A	3,1
				DNMT3A	1,2
				DNMT3A	1,4
				DNMT3A	1,6
61	M	Proximal bilateral	No	DNMT3A DNMT3A	12,2
					1,2
65	F	Proximal bilateral	Proximal	DNMT3A DNMT3A	1,4
					11,4
63	M	Segmental bilateral	No	DNMT3A	3,5
54	M	Proximal bilateral	Proximal	ASXL1	12
65	M	Segmental bilateral	No	DNMT3A	26,1

Abbreviations: CHIP, Clonal hematopoiesis of inderdermined significance; DVT, Deep Vein Thrombosis; PE, Pulmonary Embolism; VAF, Variation allelic fraction.

**Table 2:** Comparison between CHIP carriers and non carriers

	CHIP carriers	CHIP non carriers	p
Median age, IQR, y	59.5 [56.25 - 65]	54 [46 - 58]	0.08
Sex ratio, %	16	12	0.64
PE location (proximal), %	33	45	0.54
DVT proportion, %	42	47	0.66
Median hematocrit, IQR, %	0.42 [39.5–43.5]	0.43 [38–44.3]	0.61
Median platelet numeration, IQR, 10 ^9^ /L	214 [175 - 246]	207 [176 - 298]	0.55
Median WBC, IQR, 10 ^9^ /L	6.5 [5.7 - 8]	5 [3.5 - 7]	0.46

Abbreviations: CHIP, Clonal hematopoiesis of indetermined significance; DVT, Deep Vein Thrombosis; IQR, InterQuartile Range; PE, Pulmonary Embolism; WBC, White Blood Count.

Among the 19 mutations detected, 15 were found in *DNMT3A* gene, 3 in *ASXL1* and one in *TET2* . No mutation in *SF3B1, TP53, JAK2, MPL* and *CALR* were identified. 13 identified mutations were previously reported in association with hematological malignancies. The 6 other mutations were not previously reported and were considered pathologic, according to their type (frameshift mutation in *TET2, DNMT3A* and *ASXL1* ) and/or localization (same amino acid reported with a pathologic mutation involved in hematological malignancies in *DNMT3A* ).

Eight patients (66.7%) carried a unique mutation, three (25%) carried 2 mutations and one (8%) carried 5 mutations. All multiple mutations were localized in the *DNMT3A* gene, in one case associated with a *TET2* mutation.

## Discussion

Our results identify a proportion of 20% IC95%[0.1 - 0.3] of CHIP in patients with iPE. This result is nearly 4-fold higher than the expected prevalence in the general population of comparable age, considering minimal VAF of 1%. 9 Indeed, Jaiswal and al demonstrated that CHIP prevalence was found under 5,6% before 70 years of age. However, the VAF threshold used in this report was higher, at 2%. Using this 2% threshold, we still find a proportion of 10% of CHIP, representing a 75% increase in comparison to the expected rate according to published data. Therefore, CHIP could represent a clue to pathophysiopathology in iPE, becoming a new easily identifiable risk factor for PE. In this case, PE would be then no more classified as unprovoked but provoked by a permanent risk factor and then unquestionably needing long term anticoagulation.

As CHIP may induce inflammation of vascular endothelium, as recently well documented in *TET2* , 19 it could explain how vascular inflammation leads to clinical PE and also be a missing link between arterial and venous thrombosis, suspected but not yet explored. 20 As CHIP seems to be involved in many pathological conditions, previously considered as idiopathic, our results open a large amount of future clinical research.

If promising because of the careful examination and follow-up of our population, our results are limited by the small sample size, the absence of control group that doesn't allow strong statistical analysis. A second limit is the restricted number of genes detected in our panel. Various genes have been described in CHIP definition: *DNMT3A, TET2* and *ASXL1* being from far the most frequent ones according to Genovese or Jaiswal et al. 7 19 We deliberately chose to detect only 5 genes, representing altogether nearly the three quarter of mutations according to Jaiswal et al. 8 As a consequence, we may have underestimated the CHIP frequency in iPE, which may be higher if detected with a complete panel of 74 and 65 genes as tested by Jaiswal and Genovese respectively. 8 21 We chose *DNMT3A* , *TET2* and *ASXL1* since (i) they are from far the most frequent ones according to Genovese or Jaiswal et al. 7 19 and (ii) they are all involved in epigenetic regulation, which has been associated with CHIP-related premature atherosclerosis. 8 Indeed, CHIP-induced inflammation of vascular endothelium, well documented for *TET2* , 19 leading to atherosclerosis and potentially clinical IPE, may represent the missing link between arterial and venous thrombosis.

In summary, we report for the first time, the presence of high rates of CHIP in patients presenting with idiopathic pulmonary embolism. Thus, CHIP may be a new risk factor for VTE. These results need to be confirmed in an ongoing prospective case-control study including more patients and using a more diverse gene panel to better determine CHIP incidence in iPE. Moreover, whether thromboembolic risk is associated with mutations in specific genes such as *DNMT3A, ASXL1* and *TET2* , and whether these patients are exposed to a higher risk of recurrence are important questions that need to be addressed in large multicentric series.

## Notes

**What is Known on this Topic?**

- Unprovoked pulmonary embolism (uPE), defined by a proximal pulmonary embolism (PE) in the absence of major or minor risk factors, is frequent, accounting for half of reported PEs.
- Clonal hematopoiesis of indeterminate potential (CHIP) is defined by the acquisition of somatic mutations that drive clonal expansion in the absence of cytopenia, and is found in 5% of the population above 65 years.
- CHIP is associated with an increase of cardiovascular risk independently of others risk factors, related to pro-inflammatory interactions between clonally derived leukocytes and vascular endothelial models.

**What does this Paper Add?**

- Upon 61 patients without risk factors, somatic mutations of clonal hematoipoiesis were found in 12 patients (20%).
- There was no diference in terms of age, PE location and risk stratification in CHIP carriers and non carriers.
- Clonal hematoipoiesis of indeterminate potential may be a new risk factor for unprovoked pulmonary embolism.
